# Epileptic Seizure Detection Using Geometric Features Extracted from SODP Shape of EEG Signals and AsyLnCPSO-GA

**DOI:** 10.3390/e24111540

**Published:** 2022-10-26

**Authors:** Ruofan Wang, Haodong Wang, Lianshuan Shi, Chunxiao Han, Yanqiu Che

**Affiliations:** 1 School of Information Technology Engineering, Tianjin University of Technology and Education, Tianjin 300222, China; 2 Tianjin Key Laboratory of Information Sensing & Intelligent Control, School of Automation and Electrical Engineering, Tianjin University of Technology and Education, Tianjin 300222, China

**Keywords:** epileptic seizure detection, electroencephalography, second-order differential plot, geometric features, AsyLnCPSO-GA, feature selection, classification

## Abstract

Epilepsy is a neurological disorder that is characterized by transient and unexpected electrical disturbance of the brain. Seizure detection by electroencephalogram (EEG) is associated with the primary interest of the evaluation and auxiliary diagnosis of epileptic patients. The aim of this study is to establish a hybrid model with improved particle swarm optimization (PSO) and a genetic algorithm (GA) to determine the optimal combination of features for epileptic seizure detection. First, the second-order difference plot (SODP) method was applied, and ten geometric features of epileptic EEG signals were derived in each frequency band (δ, θ, α and β), forming a high-dimensional feature vector. Secondly, an optimization algorithm, AsyLnCPSO-GA, combining a modified PSO with asynchronous learning factor (AsyLnCPSO) and the genetic algorithm (GA) was proposed for feature selection. Finally, the feature combinations were fed to a naïve Bayesian classifier for epileptic seizure and seizure-free identification. The method proposed in this paper achieved 95.35% classification accuracy with a tenfold cross-validation strategy when the interfrequency bands were crossed, serving as an effective method for epilepsy detection, which could help clinicians to expeditiously diagnose epilepsy based on SODP analysis and an optimization algorithm for feature selection.

## 1. Introduction

Epilepsy is a chronic neurological disease characterized by unusual behavior, sensations and loss of awareness [1]. According to the latest epidemiological data, 65 million people worldwide are affected by epilepsy [2,3], among which approximately 30% of patients cannot be controlled with anticonvulsants and surgery [4]. Epileptic seizures can cause permanent damage to the patient’s brain, which can be monitored and detected by scalp electroencephalography (EEG) [4,5,6]. In the seizure state, scalp EEG shows a drastic increase in amplitude, with sharp wave, spike–wave, or spike (or sharp) slow wave complexes [7]. Empirically, neurophysiologists visually examine EEG signals to detect epileptic seizures. Manual monitoring of long-duration EEG signals is a monotonous and tedious job [8]. Hence, the design and development of automated epileptic seizure detection methods is considered an active field of interest for research [5,6].

A variety of modern nonlinear analysis methods have been widely used for epileptic seizure detection using EEG, such as the Lyapunov exponent [9], correlation dimension [10], complexity [11], entropy [12], fractal dimension [13] and phase space reconstruction methods. With the phase space reconstruction method, the original system is transformed into a high-dimensional system [14,15], and more information, including correlation and chaotic nonlinear dynamic characteristics of the EEG signal, can be explored in 2D projection [16]. However, phase space reconstruction is complex and time-consuming because it is dependent on the delay time parameter (τ) and the embedding dimension (d), which are computed from the input signal by mutual information and false nearest-neighbor methods [17].

Compared with phase space reconstruction, in the second-order difference plot (SODP) method, graphical representation of successive rates are compared to provide the data variability rate, which can quantify the complexity of EEG in 2D space [18]; therefore, SODP is less complex than phase space reconstruction, and it has been reported to be useful in distinguishing between various neurological disorders. Abdulhay et al. applied SODP to extract an area feature matrix for the recognition of autism spectrum disorder with 94.4% accuracy [19]. For epileptic detection, the SODP features of the shortest distance to the 45/135-degree lines (SHD), central tendency measure (CTM) [20] and the 95% confidence ellipse area [21] have been effectively verified. Most existing literature studies were based on a single SODP feature without exploring the full potential information. In [22,23,24], Wang et al. combined multiple types of features to characterize different biophysical information, improving the automatic diagnosis of neurological diseases. Hence, combining features could be useful for epileptic seizure detection.

However, the extracted features are not always capable of classifying pattern classes with absolute accuracy as the number of features increases [25]. Instead, feature classification accuracy is related to (i) highly correlated features, which may lead to redundancy in the classification learning model or (ii) uncorrelated features, which may lead to the failure of pattern recognition [26]. Therefore, it is necessary to determine the key features among a large feature set based on feature selection by intelligent optimization algorithms.

Particle swarm optimization (PSO), which was proposed by Eberhart and Kennedy in 1995, is one a well-known metaheuristic evolutionary algorithm. Inspired by the social behavior of bird flocking and fish schooling, PSO can characterize the dynamics of complex systems [27]. In the search space, a position is assigned to each particle to analyze for the optimal solution. Particle swarms find the optimal regions of the complex search space through the interaction of individuals in the population. PSO has the advantages of easy implementation and few parameters to be adjusted, but it easily falls into local extreme points [28], resulting in poor performance in the feature selection of EEG [24]. Several strategies have been proposed to improve the performance of PSO by adjusting the learning factors or inertia weights with asynchronous or synchronous changes in the learning factors, increasing the inertial weights, randomizing the inertial weights, linearly decreasing the weights, etc. Jiang et al. compared the asynchronous learning factor changes of PSO (AsyLnCPSO) with the remaining three PSO algorithms and found that AsyLnCPSO achieved the best performance in searching for a global optimum [29].

A genetic algorithm (GA) is a kind of global probabilistic search method that simulates genetic selection and natural elimination [30]. Its main characteristics are a population group search strategy and information exchange between individuals within the population. Neither relies on gradient information nor requires the solution function to be differentiable, which is available when the objective function is solvable under given constraints. Owing to its excellent scalability, it can be combined with other algorithms; thus, it has been used for feature selection in a variety of domains, such as emotional stress state detection [31], finger movement discrimination using EEG signals [32] and optimization of the kernel parameters of support vector machine (SVM) [33].

In this work, the abnormalities of EEG signals from epileptic patients are assessed based on SODP analysis, and multiple efficient geometric features are extracted to detect epileptic seizures. Then, feature selection is implemented via the proposed AsyLnCPSO-GA algorithm. Owing to the introduction of GA, AsyLnCPSO-GA can intelligently adjust the evolution of the population during optimization, increasing the robustness of the algorithm and improving the optimization accuracy compared to GA, PSO and AsyLnCPSO.

The remainder of this paper is organized as follows. In Section 2, the CHB-MIT dataset and data preprocessing are described. In Section 3, the proposed method for seizure detection is illustrated, including the SODP, geometric feature extraction, AsyLnCPSO-GA and application of AsyLnCPSO-GA in feature selection. In Section 4, the analytical results are presented, comprising analysis of SODP, statistical analysis of features, classification analysis of features and the application analysis of AsyLnCPSO-GA, followed by a discussion in Section 5 and concluding remarks in Section 6.

## 2. Data Description and Preprocessing

In this study, the Boston Children’s Hospital and the Massachusetts Institute of Technology (CHB-MIT) (https://archive.physionet.org/physiobank/database/chbmit/ (accessed on 15 July 2022)) scalp EEG dataset was used. The database contains data on 23 subjects. The sampling frequency was 256 Hz. The output of each channel was the difference in potential between electrodes. For the sake of uniformity, 23 EEG channels were selected (FP1-F7, F7-T7, T7-P7, P7-O1, FP1-F3, F3-C3, C3-P3, P3-O1, Fz-Cz, CZ-Pz, FP2-F4, F4-C4, C4-P4, P4-O2, Fp2-f8, F8-T8, T8-O2, P7-T7, T7-FT9, FT9-FT10, FT10-T8 and T8-P8), and for each channel, 4925 s of seizure data were intercepted.

**Figure 1:**
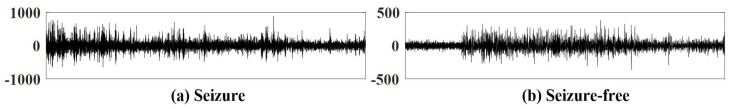
Examples of (**a**) seizure and (**b**) seizure-free EEG signals in the channel (i.e., FP1-F7).

The EEGs of the FP1-F7 channel of seizure and seizure-free cases were shown in Figure 1. In general, the investigated scalp EEG recordings contain artifacts that could deteriorate the detector performance. Therefore, artifacts caused by eye movement, muscle movements or other factors were manually removed based on a thorough offline visual inspection. To achieve high confidence in the data, the EEGs were split into segments using a sliding 20 s window with 15 s overlap to increase the sample size to a total of 327 seizure examples. Then, each channel of intercepted EEG data was decomposed into the four EEG sub-bands of interest: delta (0–4 Hz, δ), theta (4–8 Hz, θ), alpha (8–15 Hz, α) and beta (15–30 Hz, β) via a bandpass FIR filter. Moreover, the digitized EEG data were processed and analyzed in a MATLAB environment (version 9.11.0.1769968, R2021b).

## 3. Method

### 3.1. Second-Order Differential Plot (SODP)

The second-order differential plot is a graphical representation of a continuous rate of mutual contrast, which, to some extent, indicates the rate at which the signal varies. The SODP graph of the EEG signal can be obtained by plotting *x*(*i*) versus *y*(*i*), which is defined as [20,21,34,35]:

(1) x(i)=EEG(i+1)−EEG(i), y(i)=EEG(i+2)−EEG(i+1)

### 3.2. Feature Extraction

#### 3.2.1. Standard Descriptors (STDs)

**Figure 2:**
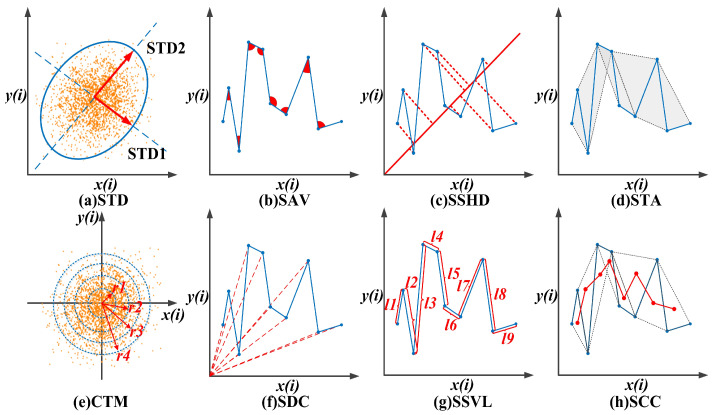
Illustration of SODP geometric features: (**a**) STD, (**b**) SAV, (**c**) SSHD, (**d**) STA, (**e**) CTM, (**f**) SDC, (**g**) SSVL, (**h**) SCC.

STDs are used to fit the ellipse and measure the dispersion and scattering of points along the minor (STD1) and major (STD2) axes [36]. STD1 and STD2 are hypothesized as two lines of 45 and 135 degrees (Figure 2a), which can be defined as follows:

(2) $$STD1=\sqrt{Var(\frac{x(i)-y(i)}{\sqrt{2}})},\;STD2=\sqrt{Var(\frac{x(i)+y(i)}{\sqrt{2}})}$$

(3) STD=π(STD1×STD2)

#### 3.2.2. Sum of the Angles between Consecutive Vectors (SAV)

The angles between successive vectors can indicate information related to EEG signal changes over time, which can quantify the behavioral complexity of the SODP in the time domain (Figure 2b). The sum of the angles between consecutive vectors is calculated as follows [37]:

(4) $$SAV=\sum_{i=1}^{n-2}\frac{x(i)\times x(i+1)+y(i)\times y(i+1)}{\sqrt{x{\left(i\right)}^{2}+y{\left(i\right)}^{2}}+\sqrt{x{\left(i+1\right)}^{2}+y{\left(i+1\right)}^{2}}}$$

#### 3.2.3. Sum of the Shortest Distance of Each Point from the 45-Degree Line (SSHD)

The shortest distance of each point from the 45-degree line (SHD) is calculated to evaluate the scattering of points on the *y = x* line (Figure 2c). The sum of the SHD can be defined as follows [38]:

(5) $$SSHD=\sum_{i=1}^{n-2}\frac{\left|x(i)-y(i)\right|}{\sqrt{2}}$$

#### 3.2.4. Sum of the Triangle Area Using Consecutive Vectors (STA)

According to SAV, consecutive three points generate angles, which can form a triangle (Figure 2d). Moreover, if the angle of the vector and the area of the generated triangle are both very small, then the distance between the consecutive points will be short, which indicates a reduction in the system dynamics to some extent [31]. Thus, the area of a triangle using consecutive vectors can be calculated as follows [34,39]:

(6) $$STA=\sum_{i=1}^{n-2}\frac{1}{2}\left|\det\left[\begin{array}{ccc} x(i) & x(i+1) & x(i+2)\\ y(i) & y(i+1) & y(i+2)\\ 1 & 1 & 1 \end{array}\right]\right|$$

#### 3.2.5. Central Tendency Measure (CTM)

The continuous-time matrices of the selected stable, non-random circular region around the origin of the SODP are computed to measure the degree of variability in the SODP plot (Figure 2e). CTM represents the number of points occupied by the SODP plot, so a low CTM value indicates that the plot data are spread over a large area. The CTM is defined as follows [20,35]:

(7) $$CTM=\frac{1}{n}\sum_{i=1}^{n}q(b_{i})$$

(8) $$q(b_{i})=\{\begin{array}{cc} 1 & if\sqrt{x{\left(i\right)}^{2}+y{\left(i\right)}^{2}}\leq r\\ 0 & otherwise \end{array}$$

where r is the radius of CTM. In this paper, the radius is set to 30–50% of the SODP range, from which three features of CTM (CTM-0.5, CTM-0.4 and CTM-0.5) are extracted.

#### 3.2.6. Sum of Distances to Coordinate (SDC)

The sum of the distance of points is computed to determine the overall scattering of SODP points on the coordinate axes (Figure 2f). It can be calculated as follows [39]:

(9) $$SDC=\sum_{i=1}^{n-2}\sqrt{x{\left(i\right)}^{2}+y{\left(i\right)}^{2}}$$

#### 3.2.7. Sum Successive of Vectors Length (SSVL)

The sum of the lengths of successive vectors (*x*(*i*), *y*(*i*)) and (*x*(*i* + *1*), *y*(*i* + *1*)) generated by successive points on the two-dimensional projection in the SODP is calculated to quantify EEG amplitude changes in the time domain (Figure 2g), which can be defined as follows [38]:

(10) $$SSVL=\sum_{i=1}^{n-1}\sqrt{{\left(x(i+1)-x(i)\right)}^{2}+{\left(y(i+1)-y(i)\right)}^{2}}$$

#### 3.2.8. Sum of the Centroid-to-Centroid Distance of Successive Triangles (SCC)

Centroids can be obtained by calculating the mean coordinates of three successive points (*x*(*i*), *y*(*i*)), (*x*(*i* + *1*), *y*(*i* + *1*)) and (*x*(*i* + *2*), *y*(*i* + *2*)); then, the distance between the centroids of every two successive triangles is computed to quantify the self-similarity of the SODP (Figure 2h). The sum of centroid-to-centroid distance can be defined as follows [38]:

(11) $$x_{C_{i}}=\frac{x(i)+x(i-1)+x(i-2)}{3},\;y_{C_{i}}=\frac{y(i)+y(i-1)+y(i-2)}{3}$$

(12) $$SCC=\sum_{i=3}^{n}\sqrt{{\left(x_{C_{i+1}}-x_{C_{i}}\right)}^{2}+{\left(y_{C_{i+1}}-y_{C_{i}}\right)}^{2}}$$

### 3.3. AsyLnCPSO-GA 

**Figure 3:**
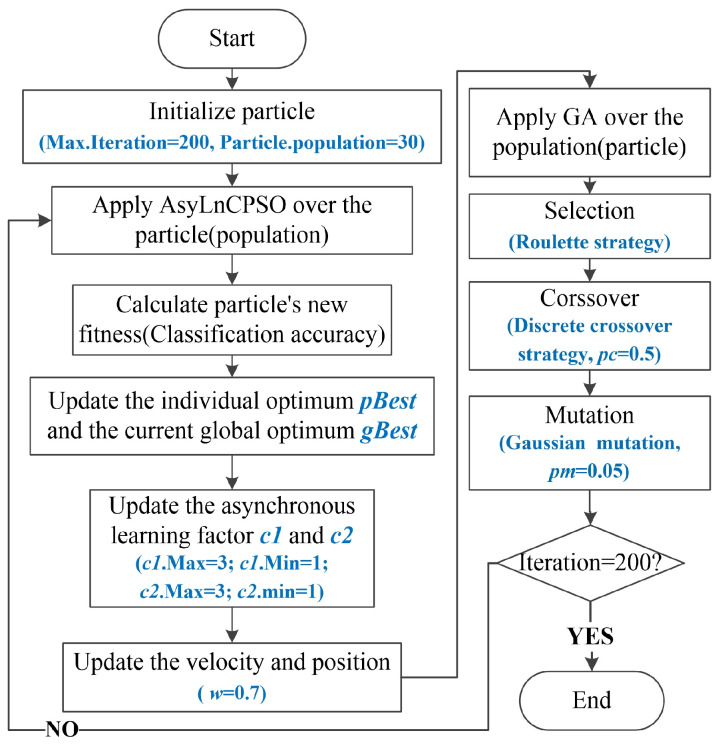
Flow chart of AsyLnCPSO-GA.

In this paper, we propose an improved optimization algorithm, AsyLnCPSO-GA, which combines AsyLnCPSO with the genetic algorithm (GA). In each iteration, particles are first optimized by AsyLnCPSO; then, all optimized particles are fed to GA to prevent some particles from becoming trapped in local optimization. The flow chart of the proposed algorithm is shown in Figure 3. Here, the initial particle swarm size is set to 30, the number of iterations is 200 and the trials are carried out 20 times. Moreover, computation complexity of AsyLnCPSO-GA in each iteration is: (1) AsyLnCPSO: O(Position Update × Particle size) + O(Velocity Update Particle size) + O(Fitness Calculation × Particle size), (2) GA: O((Crossover + Fitness Calculation) × (Crossover-rate × Particle size)) + O((Mutation + Fitness Calculation) × (Mutation-rate × Particle size)). Detailed descriptions of the GA, PSO and AsyLnCPSO algorithms are provided in Appendix A.

### 3.4. Application of AsyLnCPSO-GA in Feature Selection

**Figure 4:**
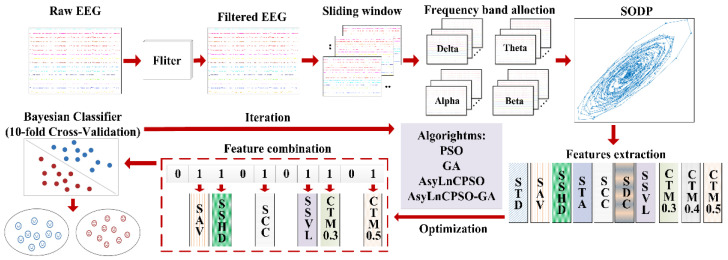
Intelligent process for epileptic seizure detection.

The application process of AsyLnCPSO-GA is as follows: First, the features are sorted according to the following sequence: STD, SAV, SDC, STA, SSHD, SCC, SSVL and CTM (CTM-0.3~CTM-0.5), which are randomly combined and represented as algorithm particles. Each particle is composed of 0–1 sites with a length of 10–40 bits (single-band: 10 bits, dual-band: 20 bits, three-band: 30 bits and four-band: 40 bits), where the number 1 indicates that the feature is selected for input to the classifier and vice versa. For example, suppose that the feature combination in the single-band setup is represented by the particle [0100100001 (δ)]; accordingly, the features SAV (2nd), SSHD (5th) and CTM-0.5 (10th) are selected for combination. Secondly, the Bayesian classifier is chosen as the fitness function of the algorithm, and the classification accuracies of feature combinations are determined as the fitness values. In the model of the Bayesian classifier, 10-fold cross validation is applied, and the ratio of training to test data is 9:1. Owing to the real value of the algorithm, the real values of the particles need to be transferred into to 0–1 by threshold τ(τ=0) before being input into the Bayesian classifier. The intelligent detection process is shown in Figure 4.

## 4. Results

### 4.1. Analysis of SODP

**Figure 5:**
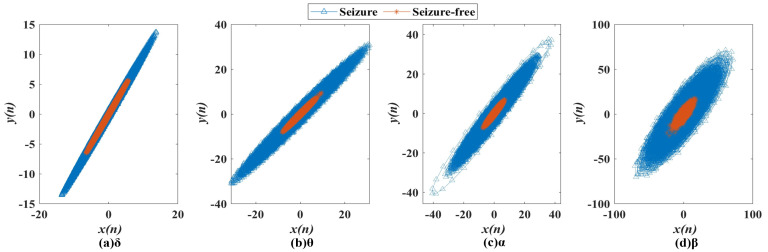
SODP of seizure (blue line) and seizure-free (red line) groups in the (**a**) δ, (**b**) θ, (**c**) α and (**d**) β frequency bands.

In order to study the abnormal fluctuations of EEG in the δ, θ, α and β frequency bands of epileptic patients, the SODP graph composed of *x*(*n*) and *y*(*n*) was generated, as shown in Figure 5, where every two consecutive dots are indicated by connecting lines: the blue line represents epileptic seizures, and the red line represents seizure-free data. According to Figure 5, the SODP of epileptic seizure EEG occupies significantly more areas than the seizure-free data, which is associated with the abnormal discharge of epileptic seizures. In particular, the values of epileptic seizure group were in the ranged of [−13.58, 13.71] in the δ frequency band, [−30.83, 31.15] in the θ frequency band, [−40.52, 37.76] in the α frequency band and [−70.26, 71.19] in the β frequency band, while in the seizure-free group, the values were in the range of [−6.51, 5.56], [−8.13, 9.46], [−7.34, 7.16] and [−24.26, 18.35] in the δ, θ, α and β frequency bands, respectively. In the four frequency bands, the SODP of seizure and seizure-free data showed obvious differences. The more obvious the difference, the closer the distribution of the SODP graph to *y = x*, suggesting that the abnormal fluctuation of epileptic EEG was discontinuous intermittent abnormal discharge.

### 4.2. Statistical Analysis of Features

**Figure 6:**
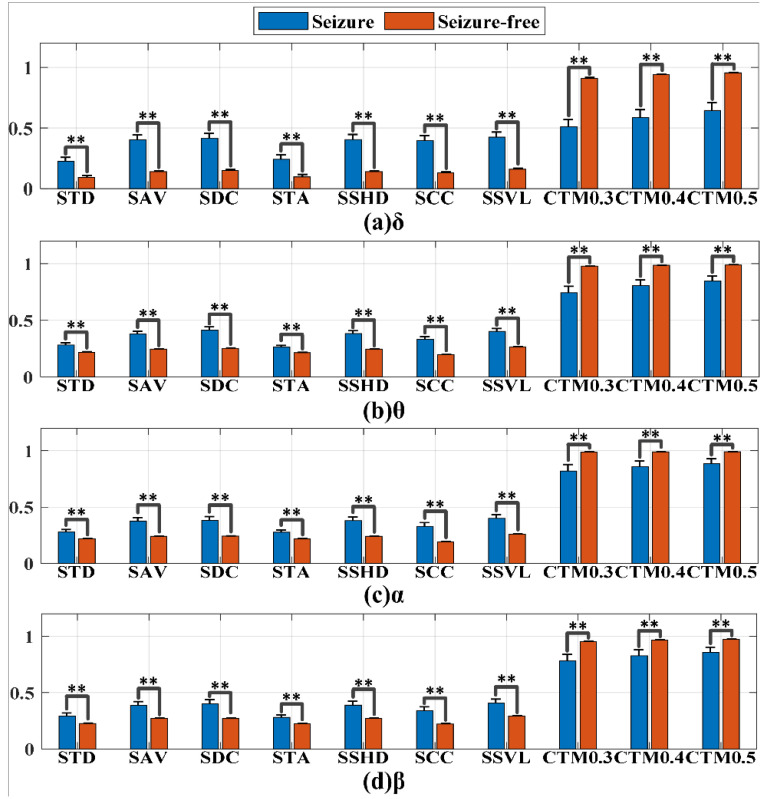
Feature (normalized in the range of [0, 1]) visualization in the (**a**) δ, (**b**) θ, (**c**) α and (**d**) β frequency bands. Asterisks represent significant differences between the two groups (“**”: *p* < 0.01).

Ten geometric nonlinear features were extracted: STD, SAV, SDC, STA, SSHD, SCC, SSVL, CTM-0.3, CTM-0.4 and CTM-0.5. Given the differences in the ranges of the four frequency bands, the radiuses of the CTM features were expressed as the proportion of the SODP ranges. One-way ANOVA statistical analysis was applied to the ten SODP features to assess the difference between the epileptic seizure and seizure-free groups, as shown in Figure 6, where asterisks represent significant differences between the two groups (“**”: *p* < 0.01). Here, the values were normalized in the range of [0,1] for the convenience of display. The mean ± standard deviation and *p*-value are shown in Appendix Table A1. For the first seven features, the values increased in the seizure group, whereas for the last three CTM features, the values decreased. The increase in the former seven features in the epileptic seizure group indicates that the SODP extends in the y = x direction, that scatters from the coordinate center were wide, the triangle area of three continuous points and the distance of continuous triangle centroid in SODP were large, the distance between two consecutive points was longer and the fluctuation amplitude was large, showing increased self-similarity and behavioral complexity of EEG signals. In contrast, the seizure group had lower CTM values than the seizure-free group, associated with larger SODP scatter in the seizure-free group, which was essentially consistent with the results for the former seven features, i.e., STD–SSVL. In summary, all features in the four frequency bands showed significant group differences (*p* < 0.01), which could be considered for further classification study.

### 4.3. Classification Analysis of Features

The averaged classification results are shown in Table 1. For all features, the δ band showed the best classification effect, for which the highest classification accuracy reached 0.8356 in CTM-0.3, followed by the θ band, for which the highest accuracy was 0.7822 in CTM-0.3, whereas the α and β bands had poor discrimination, with the highest classification accuracies of 0.7158 in SAV and 0.6317 in CTM-0.3, respectively. Among the ten features, CTM-0.3 performed best in the δ, θ and β bands.

### 4.4. Application Results of GA, PSO, AsyLnCPSO and AsyLnCPSO-GA

In this section, in order to improve the classification accuracy in each frequency band, multiple features were combined, and intelligent optimization algorithms (GA, PSO, AsyLnCPSO and AsyLnCPSO-GA) were applied to determine the optimal feature combination.

#### 4.4.1. Simulation Test

**Figure 7:**
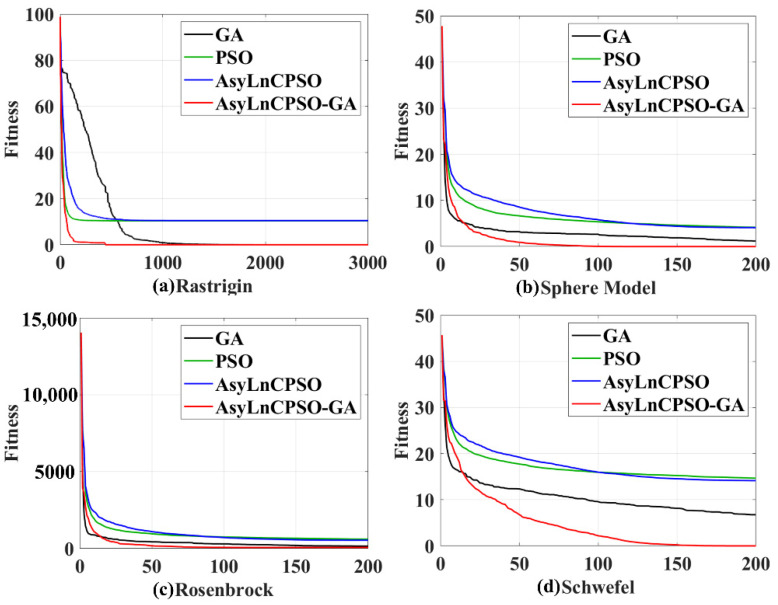
Simulation test results of the four algorithms with the (**a**) Rastrigin, (**b**) Sphere Model, (**c**) Rosenbrock and (**d**) Schwefel functions. GA: black line; PSO: green line; AsyLnCPSO: blue line; AsyLnCPSO-GA: red line.

First, intelligent algorithms were used with the Rastrigin, Sphere Mode, Rosenbrock and Schwefel functions to verify their effectiveness, and the population diversity, optimization precision, execution efficiency and capability of the global search were assessed [40]. The average values of *gBest* for the four algorithms over 20 trials were compared, as shown in Figure 7 and Table 2. All four algorithms showed the ability to optimize. The time consumption of AsyLnCPSO-GA was more than that of the other three algorithms, indicating that AsyLnCPSO-GA increased the computational complexity (Table 2), although it achieved the best performance, with a quick convergence speed in high-dimensional space, the highest optimization precision and execution efficiency and most solution spaces with the lowest fitness value (Figure 7).

#### 4.4.2. Application Analysis of Seizure Detection

**Figure 8:**
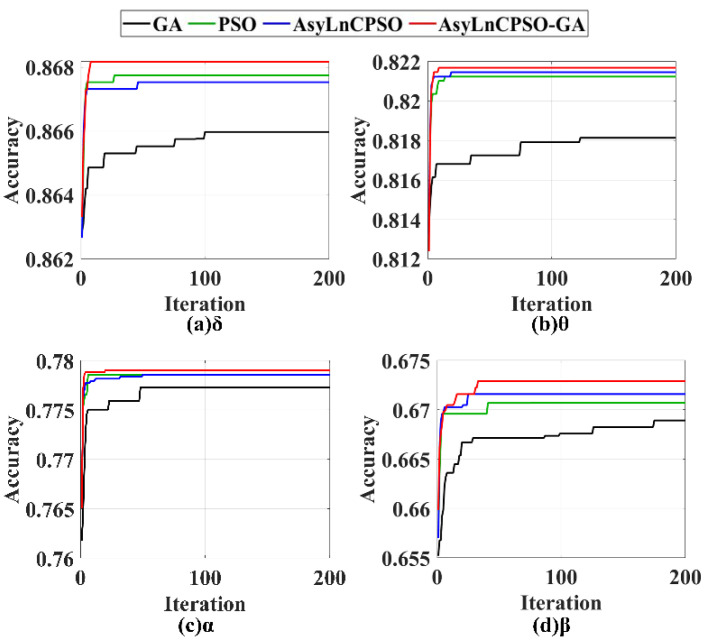
The trends of the optimal particle (*gBest*) optimized by GA, PSO, AsyLnCPSO and AsyLnCPSO-GA in the (**a**) δ, (**b**) θ, (**c**) α and (**d**) β frequency bands. GA: black line; PSO: green line; AsyLnCPSO: blue line; AsyLnCPSO: red line.

The features were combined in each frequency band, and GA, PSO, AsyLnCPSO and AsyLnCPSO-GA were applied to determine the optimal feature combination. A total of 20 experimental trials were conducted with each algorithm, and the trends in the classification accuracies of the best feature combinations (*gBest*) optimized by GA, PSO, AsyLnCPSO and AsyLnCPSO-GA with an increased in the number of iterations are exhibited in Figure 8, where the horizontal axis represents the number of iterations, and the vertical axis represents the classification accuracies of the *gBest.* GA, PSO, AsyLnCPSO and AsyLnCPSO-GA are plotted as black, green, blue and red lines, respectively. The higher the value, the better the optimization ability of the algorithm. All four algorithms were able to optimize the features, but AsyLnCPSO-GA achieved the best performance, with fast convergence, small fluctuations and the highest classification accuracy. Furthermore, the lowest, highest and average classification accuracies were calculated, as shown in Table 3. First, compared to Table 1, the classification accuracies obtained with the combinations of features by the optimization algorithms were higher than those obtained with a single feature in each frequency band. For instance, in the δ frequency band, the classification accuracies of feature combinations were 0.8660, 0.8677, 0.8675 and 0.8682—all higher than the 0.8356 obtained with CTM-0.3 only. Secondly, similar to the results presented in Table 1, the classification effects of the δ and θ bands were better than those of α and β bands. Finally, AsyLnCPSO-GA considerably improved the classification and achieved the highest average accuracy in each frequency band.

**Figure 9:**
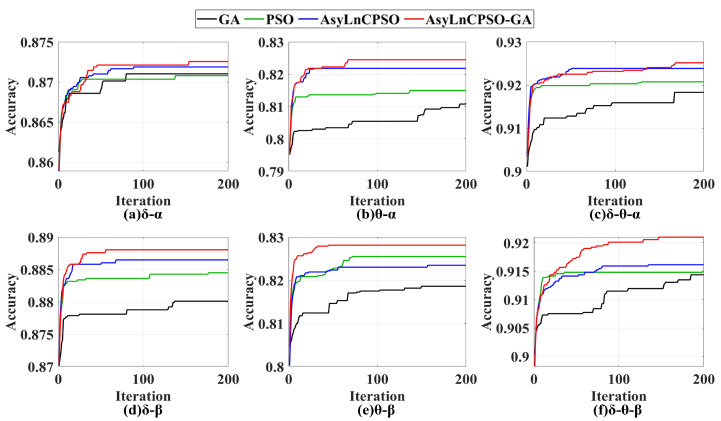
Trends of optimal particles (*gBest*) optimized by the GA, PSO, AsyLnCPSO and AsyLnCPSO-GA algorithms in the (**a**) δ-α, (**b**) θ-α, (**c**) δ-θ-α, (**d**) δ-β, (**e**) θ-β and (**f**) δ-θ-β bands. GA: black line; PSO: green line; AsyLnCPSO: blue line; AsyLnCPSO: red line.

To further investigate the influence of the δ and θ bands on the accuracies of the α and β bands, the features from different bands were combined (i) by δ-α, θ-α and δ-θ-α to analyze the effect of δ and θ on the α frequency band; (ii) by δ-β, θ-β and δ-θ-β to analyze the effect of δ and θ on the β frequency band. As shown in Figure 9 and Table 4, the average accuracies of AsyLnCPSO-GA were better than those of the other three algorithms (Figure 9), which is consistent with the results presented in Figure 8. Compared with the results shown in Table 3, the introduction of δ or θ bands in band crossing had a significant impact on the classification effect in single α or β bands, e.g., the maximal classification accuracies of δ-α and θ-α were 0.8726 and 0.8245 higher than the 0.7790 accuracy achieved with the α band alone (Table 3), with the same result with respect to the β band. Additionally, the simultaneous introduction of two crossing frequency bands (δ-θ) improved the classification effect, e.g., the classification results of three bands (δ-θ-α and δ-θ-β) were 0.9252 and 0.9210, respectively—higher than that achieved dual bands.

**Figure 10:**
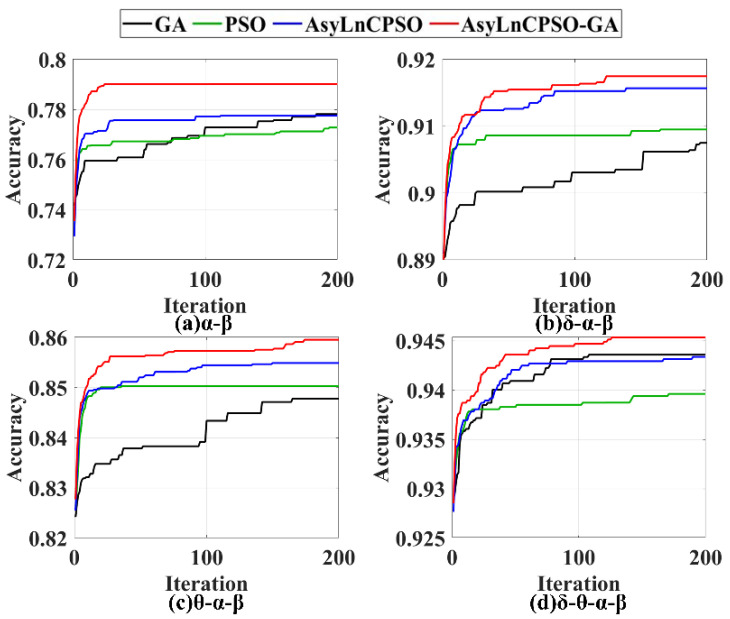
The trends of the optimal particles (*gBest*) optimized by the GA, PSO, AsyLnCPSO and AsyLnCPSO-GA algorithms in the (**a**) α-β, (**b**) δ-α-β, (**c**) θ-α-β and (**d**) δ-θ-α-β bands. GA: black line; PSO: green line; AsyLnCPSO: blue line; AsyLnCPSO: red line.

Subsequently, the frequency bands (α-β, δ-α-β, θ-α-β and δ-θ-α-β) were crossed to study the effect of δ and θ on the cross-frequency band (α-β), as shown in Figure 10 and Table 5. The introduction of the combination of superior bands (δ/θ/δ-θ) considerably improved the classification effect of inferior crossing bands (α-β), e.g., the average accuracies of δ-α-β, θ-α-β and δ-θ-α-β were increased to 0.9175, 0.8595 and 0.9454, respectively—all higher than the 0.7901 accuracy of α-β (Table 5). Notably, the AsyLnCPSO-GA algorithm achieved the best performance among the four algorithms; for instance, in the four-band crossing situation (δ-θ-α-β), the highest average accuracies of the GA, PSO, AsyLnCPSO and AsyLnCPSO-GA algorithms were 0.9436, 0.9396, 0.9434 and 0.9454, respectively.

As shown in Figure 8, Figure 9 and Figure 10, PSO and AsyLnCPSO fell into local optima with a low accuracy, whereas GA converged slowly and fluctuated as a result of the introduction of a mutation operator. However, with the introduction of GA into AsyLnCPSO, the combined AsyLnCPSO-GA algorithm improved the global optimal search ability and screened the best feature combination much faster and more accurately.

Finally, the average time consumption and feature dimension during optimization were calculated, as shown in Table 6. AsyLnCPSO presented the longest running time from the single-band to four-band crossover, whereas GA had the shortest running time, with crossover and mutation rates of 0.5 and 0.01, respectively, resulting in GA calculating the fitness of more than half of the particles in each iteration. The average feature dimension of the AsyLnCPSO-GA algorithm optimized in the three-band and four-band crossovers was more than that of the other three algorithms, suggesting that AsyLnCPSO-GA could search a much wider search space (high-dimensional space) to avoid falling into the local optimum, which is consistent with the results presented in Figure 7.

#### 4.4.3. Analysis of Key Features

Accuracy is often improved as the number of features is increased and features are combined. However, some combinations with few features, called key features, can achieve high classification accuracy, reduce the dimension of the feature vector and preserve the most important information. Thus, in order to investigate the effect of the key features on classification accuracy, statistical analysis of the occurrence frequency of key features was conducted in the 15 classes of interband combinations (δ, θ, α, β, δ-θ, δ-α, δ-β, θ-α, θ-β, α-β, δ-θ-α, δ-θ-β, δ-α-β, θ-α-β, δ-θ-α-β), and the top four features with the most occurrences were selected, as shown in Table 7. First, CTM features occurred most frequently and became the key features in the single-frequency band, i.e., CTM-0.3: 75% in the δ band, CTM-0.4: 100% in the θ band, CTM-0.3: 60% in the α band and CTM-0.4: 95% in the β band. However, other features appeared more than the CTM features when frequency bands crossed, e.g., SAV, SSH and STD in the δ- combinations (“δ-” represents that the band combinations contained the δ frequency band); SAV, SSHD and SSVL in the θ- combinations; STD, SAV, SCC and SSHD in the α- combinations; and STD, SDC, SSVL, SAV and SSHD in the β- combinations. As mentioned above, the key features were differed depending on the frequency band crossing; therefore, it was necessary to automatically determine the key features by using the intelligence optimization algorithm.

**Figure 11:**
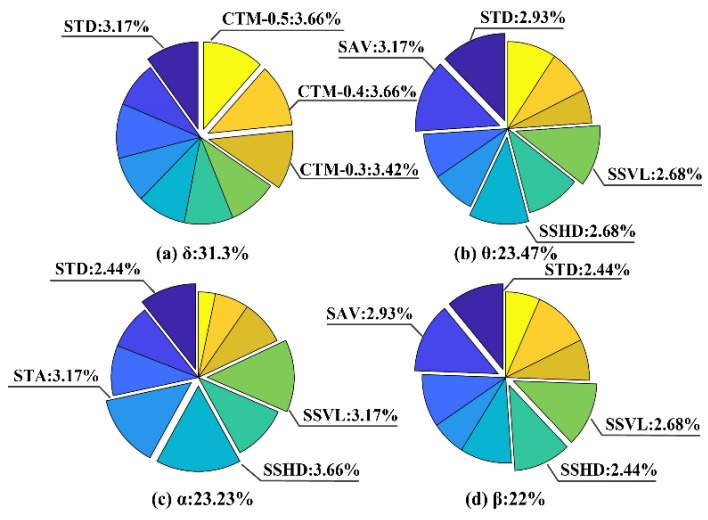
The total percentage of the appearance of features in the (**a**) δ, (**b**) θ, (**c**) α and (**d**) β frequency bands.

Finally, the band crossing δ-θ-α-β was adopted to investigate (i) the impacts of different bands on δ-θ-α-β and (ii) the distribution difference of key features in each constituent frequency band of δ-θ-α-β. As shown in Figure 11, (i) the percentage of the δ frequency band was 31.3%, which was the highest, followed by the θ frequency band (23.57%), the α frequency band (23.23%) and the β frequency band (22%), suggesting that the α and β frequency bands with poor classification could also contribute to improvement in the classification accuracy in the interband crossing. (ii) The distribution of the top four key features differed in each constituent frequency band: CTM-0.5, 3.66%; CTM-0.4, 3.66%; CTM-0.3, 3.42%; and STD, 3.17% in the δ frequency band; SAV, 3.17%; STD, 2.93%; SSHD, 2.68%; and SSVL, 2.68% in the θ frequency band; SSHD, 3.66%; STA, 3.17%; SSVL, 3.17%; and SCC, 2.44% in the α frequency band; and SAV, 2.93%; SSVL, 2.68%; CTM-0.4, 2.44%; and STD, 2.44% in the β frequency band, demonstrating that the combination of key features in different frequency bands can improve seizure detection.

## 5. Discussion

In this study, epileptic seizure and seizure-free EEG signals were plotted by SODP, and ten nonlinear geometric features were extracted in each frequency band (δ, θ, α and β) to detect seizures. The results showed that the 2D SODP projection of seizure EEG signals occupied more space than seizure-free signals, indicating that it contained more rhythmic and irregular shapes. Owing to the paroxysmal abnormal firing of brain neurons, EEG signals of seizures exhibit less stationary morphological behaviors and more complex behaviors than seizure-free EEG signals [15,18,41,42], such as spikes in epileptic seizures [39], leading to sharp edges on the 2D projection [38], as shown in Figure 5, and significant group differences between seizure and seizure-free signals (Table A1 in Appendix A), especially in the δ and θ frequency bands. Thus, geometric features can be considered effective markers for seizure detection. Furthermore, in order to evaluate the effectiveness of these features, eight simple time-domain features (root mean square, peak–peak value, skewness, kurtosis, shape indicator, crest indicator, impulse indicator, and clearance indicator) were extracted in the δ frequency band for comparison with geometric features, as shown in Table A1 for geometric features and Table A2 for time-domain features. Both the time-domain and geometric features exhibited significant differences between seizure and seizure-free signals. However, most of the geometric features performed better than the time-domain features for classification, in part because the geometric features can not only express the simple information of EEG signals, such as time-domain features, but also the complexity of EEG signals in 2D space, owing to their dynamic and chaotic nature [18].

Previous studies have shown that slow waves (<4 Hz, δ frequency band) are of prime importance for the detection of focal epilepsy [43]. Tao et al. [44] proposed interictal regional δ slowing as an EEG biomarker for temporal seizure detection. Schönherr et al. [45] reported that postoperative δ activity can be used as a diagnostic marker for recurrent seizures, similar to the results reported in the present study. In contrast, in the present study, we also reported significant group differences in the θ frequency band with respect to geometric features detected by SODP that were rarely observed in previous studies. For fast waves (α and β) group differences were significantly reduced.

Most previous studies with respect to biomedical signal processing applications have employed statistical approaches, such as ANOVA and Student’s *t*-test, as feature selection tools. In other words, they used *p*-values to select significant features, i.e., features with *p*-values less than 0.05 or 0.01 were selected as salient. However, this approach is not always useful when *p*-values of all features are less than 0.01, such as the ten features extracted in this study (Figure 6). As shown in Table 1, single features cannot effectively distinguish epileptic seizure signals from seizure-free signals in the α and β frequency bands. Moreover, extracted features are not always capable of classifying the pattern classes with absolute accuracy as the number of features increases [25], largely because features are highly correlated or similar to each other, leading to redundancy in the learning model when both features are included. In contrast, features are uncorrelated with the pattern class to be predicted, i.e., the features are not useful enough to represent the pattern classes properly [26]. All features are assumed to be used in the classifier, whereby the feature vector length will have the highest length. If the feature vector excessively long, the complexity of the classifier will be extremely high. Therefore, it is necessary to screen the shortest feature vector with the best performance, and the most optimal features are considered as key features here.

In this study, to overcome the lack of exploitation ability in the genetic algorithm (GA), slow convergence, premature convergence and the tendency to fall into the local optimal solution in particle swarm optimization (PSO) [46,47,48], a novel combined method, AsyLnCPSO-GA, was presented and introduced to select the optimal feature combination, then fed to the naïve Bayesian classifier. Owing to the combination of AsyLnCPSO and GA, AsyLnCPSO-GA achieved best performance in feature selection compared with PSO and the improved PSO–AsyLnCPSO (Figure 7, Figure 8, Figure 9 and Figure 10) algorithms. AsyLnCPSO conducted a thorough search in the search space by using particles that related feature information to one another, whereas the GA performed adequately in terms of passing down useful features from one generation to the next. As a consequence, the classification accuracies of feature combination optimized by the AsyLnCPSO-GA algorithm was considerably improved, with a maximum accuracy of 0.9535 in δ-θ-α-β (Table 5). 

To increase the robustness of the results, the Kaggle [49], U-Bonn [50] and NSC-ND [51] datasets were used, demonstrating that the AsyLnCPSO-GA algorithm proposed in this paper achieved a high classification accuracy, as shown in Table 8. Comparison results (Table 8) show that the feature combinations differed depending on the optimization algorithm, indicating that in detection, it is necessary to adaptively optimize the feature combination by applying intelligent algorithms because certain fixed features might not work. Details of previous studies on epilepsy detection using these datasets are summarized in Table 9 in comparison with the framework proposed in this paper. Evidently, seizures could be detected efficiently by all the methods listed, with classification accuracies of more than 0.9, and the proposed framework outperformed several existing models [52,53,54,55,56,57,58,59,60,61,62,63,64,65]. However, few studies showed better classification results than that achieved in the present study, possibly owing to the selection of non-seizure data, the selection of data sample size, the difference in data preprocessing, the difference in the applied method applied, the difference in classifier, etc. For instance, in [52], the AUC values of the SVM classifier were 0.9432 (dog) and 0.9349 (human), whereas the Bayesian classifier reached 0.7594 (dog) and 0.7664 (human). Similarly, in [62], the accuracy of the RF classifier reached 0.9941, whereas that of the Bayesian classifier was 0.9516, which was inferior to that of the method proposed in this paper. Additionally, in [62,63], the classification in the CHB-MIT dataset was performed by using individual patients separately, resulting in decreased analysis complexity.

However, the present study is subject to some limitations. (1) The geometric features extracted in this paper are based on the SODP method, and more types of features, such as time-domain, frequency-domain, time-frequency domain features and other features, can be extracted and combined for epilepsy detection. (2) The proposed AsyLnCPSO-GA algorithm does not take into account the impact of the combining modes between the two algorithms, which could be optimized by adopting different combination strategies and fewer calculation formulae in future research work. (3) Alternative classifiers, such as SVM, KNN, RF, random forest and logistic regression, could be used to improve the classification accuracy.

## 6. Conclusions

In this paper, ten geometric features (STD–CTM) based on SODP formation patterns in EEG signals were extracted for epileptic seizure detection. Analysis of SODP in four frequency bands (δ, θ, α and β) showed that compared with the seizure-free group, the area of the SODP in the epileptic seizure group occupied significantly more space. ANOVA statistical analysis and classification analysis were further applied to assess the effectiveness of the SODP based on geometric features. Although all features in the four frequency bands differed significantly, the classification accuracies of most features generated by the Bayes classifier in the α and β frequency bands were low. In order to improve seizure detection, a novel hybrid algorithm, AsyLnCPSO-GA, was proposed for multiple feature combination (δ–δ-θ-α-β), achieving a much higher classification accuracy than the GA, PSO and AsyLnCPSO algorithms, with a maximum classification accuracy of 0.9535 in the δ-θ-α-β combination. In addition, the following results were obtained: (i) for target features in the feature combination, the impacts of key features were investigated by counting the occurrence frequency of features in all combinations, showing that the key features differed depending on the frequency band crossing, demonstrating the necessity and importance of automatically determining the key features by using the intelligence optimization algorithm; (ii) for target frequency bands in the feature combination, the introduction of the superior bands (δ/θ/δ-θ) considerably improved the classification effect of the inferior bands (α/β/α-β), whereas bands (α/β/α-β) with poor classification also contributed to improvements in classification accuracy in the interband crossings.

In summary, the hybrid model with AsyLnCPSO-GA and a naïve Bayesian classifier based on SODP shape analysis can applied to explore the potential markers and characterize the abnormalities of EEG signals of epileptic seizures, possibly shedding light on epileptic EEG analysis and extending our understanding of brain function in patients with neurological diseases.

## Figures and Tables

**Table 1:** The classification results of SODP single features (STD–CTM-0.5) in the δ, θ, α and β frequency bands.

Band	STD	SAV	SDC	STA	SSHD	SCC	SSVL	CTM-0.3	CTM-0.4	CTM-0.5
δ	0.6734	0.8284	0.8228	0.6491	0.8347	0.8252	0.8042	0.8356	0.8198	0.8011
θ	0.5758	0.7692	0.7701	0.5796	0.7557	0.7581	0.7582	0.7822	0.7509	0.6927
α	0.5712	0.7158	0.7087	0.5711	0.7062	0.7011	0.6985	0.7108	0.6523	0.6252
β	0.5693	0.6272	0.6238	0.5642	0.6102	0.6124	0.6107	0.6317	0.6093	0.5953

**Table 2:** Average time consumption and fitness values of the simulation test.

Algorithm	Test Function							
	Rastrigin		Sphere Model		Rosenbrock		Schwefel	
	Time Consumption	Fitness Value	Time Consumption	Fitness Value	Time Consumption	Fitness Value	Time Consumption	Fitness Value
GA	0.313 s	1.92 × 10^−4^	0.016 s	1.201	0.013 s	154.149	0.045 s	6.762
PSO	0.392 s	10.494	0.021 s	4.173	0.02 s	609.415	0.065 s	14.687
AsyLnCPSO	0.425 s	10.417	0.021 s	4.105	0.021 s	541.367	0.066 s	14.143
AsyLnCPSO-GA	0.671 s	0	0.035 s	2.03 × 10^−7^	0.03 s	68.287	0.094 s	1.93× 10^−3^

**Table 3:** Classification accuracies (min/max/average) optimized by GA, PSO, AsyLnCPSO and AsyLnCPSO-GA in the δ, θ, α and β bands.

	GA			PSO			AsyLnCPSO			AsyLnCPSO-GA		
	Min	Max	Avg	Min	Max	Avg	Min	Max	Avg	Min	Max	Avg
δ	0.8606	0.8719	0.8660	0.8651	0.8719	0.8677	0.8651	0.8719	0.8675	0.8651	0.8719	0.8682
θ	0.8121	0.8251	0.8181	0.8186	0.8251	0.8212	0.8187	0.8251	0.8215	0.8206	0.8251	0.8217
α	0.7698	0.7856	0.7772	0.7719	0.7878	0.7786	0.7701	0.7878	0.7786	0.7763	0.7878	0.7790
β	0.6547	0.6792	0.6689	0.6592	0.6768	0.6707	0.6638	0.6792	0.6716	0.6681	0.6792	0.6729

**Table 4:** Classification accuracies (min/max/average) optimized by the GA, PSO, AsyLnCPSO and AsyLnCPSO-GA algorithms in the δ-α, θ-α, δ-θ-α, δ-β, θ-β and δ-θ-β bands.

	GA			PSO			AsyLnCPSO			AsyLnCPSO-GA		
	Min	Max	Avg	Min	Max	Avg	Min	Max	Avg	Min	Max	Avg
δ-α	0.8627	0.8784	0.8710	0.8671	0.8739	0.8708	0.8627	0.8783	0.8719	0.8671	0.8783	0.8726
θ-α	0.7940	0.8293	0.8110	0.8052	0.8229	0.8150	0.8054	0.8343	0.8219	0.8187	0.8316	0.8245
δ-θ-α	0.9070	0.9292	0.9183	0.9114	0.9291	0.9208	0.9203	0.9271	0.9239	0.9204	0.9292	0.9252
δ-β	0.8716	0.8916	0.8801	0.8760	0.8959	0.8845	0.8760	0.8959	0.8865	0.8783	0.8959	0.8880
θ-β	0.7963	0.8320	0.8186	0.8166	0.8320	0.8255	0.8142	0.8321	0.8235	0.8185	0.8363	0.8281
δ-θ-β	0.9003	0.9271	0.9144	0.9048	0.9249	0.9150	0.9070	0.9226	0.9161	0.9138	0.9315	0.9210

**Table 5:** Classification accuracies (min/max/average) optimized by the GA, PSO, AsyLnCPSO and AsyLnCPSO-GA algorithms in the α-β, δ-α-β, θ-α-β and δ-θ-α-β bands.

	GA			PSO			AsyLnCPSO			AsyLnCPSO-GA		
	Min	Max	Avg	Min	Max	Avg	Min	Max	Avg	Min	Max	Avg
α-β	0.7593	0.7876	0.7782	0.7628	0.7900	0.7728	0.7480	0.7946	0.7775	0.7767	0.7969	0.7901
δ-α-β	0.8936	0.9250	0.9075	0.8963	0.9203	0.9095	0.9003	0.9270	0.9157	0.9026	0.9269	0.9175
θ-α-β	0.8294	0.8673	0.8478	0.8364	0.8630	0.8503	0.8517	0.8606	0.8549	0.8494	0.8718	0.8595
δ-θ-α-β	0.9314	0.9535	0.9436	0.9315	0.9425	0.9396	0.9315	0.9535	0.9434	0.9381	0.9535	0.9454

**Table 6:** The average time consumption and feature dimension of GA-, PSO-, AsyLnCPSO- and AsyLnCPSO-GA-optimized single-band, dual-band, three-band and four-band crossovers.

Algorithm (Naïve Bayesian)	Single-Band		Dual-Band		Three-Band		Four-Band	
	Time Consumption	Feature Dimension	Time Consumption	Feature Dimension	Time Consumption	Feature Dimension	Time Consumption	Feature Dimension
GA	78.14 s	6.5	91.82 s	12.4	103.21 s	16.5	125.36 s	19.2
PSO	117.28 s	4.8	144.57 s	9.9	173.5 s	13.3	198.69 s	15.3
AsyLnCPSO	121.39 s	4.9	152.23 s	10.4	187.31 s	15.1	201.54 s	18.1
AsyLnCPSO-GA	174.25 s	5.2	213.84 s	11.3	259.64	16.7	288.13 s	21.6

**Table 7:** The proportion of features (top four) in *gBest* of δ–δ-θ-α-β. Annotation: “%” is the frequency of feature occurrence in all trials.

Band	The Proportion of Features in *gBest*
δ	δ: CTM-0.3 (75%), CTM-0.4 (95%), SAV (60%%), SSHD (50%).
θ	θ: CTM-0.4 (100%), SSHD (75%), SAV (70%), CTM-0.5 (60%).
α	α: SSHD (100%), SAV (80%), CTM-0.3 (60%), STD (50%).
β	β: CTM-0.4 (95%), SAV (80%), CTM-0.3 (60%), SDC (30%).
δ-θ	δ: CTM-0.3 (100%), SSHD (85%), SAV (85%), CTM-0.4 (50%); θ: SAV (85%), CTM-0.3 (75%), STD (60%), STA (30%).
δ-α	δ: CTM-0.3 (95%), SAV (95%), SSHD (90%), SCC (40%); α: SSHD (55%), SAV (50%), CTM-0.3 (40%), STA (15%).
δ-β	δ: SSHD (95%), CTM-0.3 (90%), STD (90%), CTM-0.5 (45%); β: SSHD (90%), SAV (70%), CTM-0.3 (35%), SCC (20%).
θ-α	θ: SAV (95%), CTM-0.3 (90%), SSHD (85%), SSVL (30%); α: SSHD (60%), CTM-0.3 (20%), SDC (10%), SAV (10%).
θ-β	θ: CTM-0.3 (100%), SAV (95%), SSHD (65%), SDC (30%); β: SAV (65%), SSHD (25%), STD (5%), SCC (5%).
α-β	α: SAV (100%), SSHD (80%), CTM-0.3 (30%), SSVL (25%); β: SAV (25%), SSHD (25%), STD (10%), CTM-0.3 (10%).
δ-θ-α	δ: CTM-0.3 (80%), CTM-0.5 (40%), SAV (35%), CTM-0.4 (30%); θ: CTM-0.5 (100%), SAV (100%), SSHD (65%), STD (55%); α: SAV (60%), CTM-0.5 (55%), SSHD (50%), SSVL (25%).
δ-θ-β	δ: CTM-0.3 (70%), SAV (65%), STD (40%), CTM-0.5 (40%); θ: CTM-0.4 (100%), SAV.5 (80%), CTM-0.3 (80%), SSHD (60%); β: CTM-0.5 (65%), SAV (40%), SSHD (30%), CTM-0.3 (25%).
δ-α-β	δ: SSHD (75%), CTM-0.5 (70%), SAV (70%), STD (60%); α: SAV (100%), SSVL (85%), SSHD (80%), CTM-0.3 (65%); β: SSVL (35%), CTM-0.3 (35%), STD (35%), CTM-0.5 (35%).
θ-α-β	θ: STA (75%), CTM-0.4 (70%), CTM-0.5 (70%), SAV (65%); α: SSHD (90%), SAV (65%), CTM-0.3 (60%), CTM-0.4 (60%); β: CTM-0.4 (55%), STD (40%), SSVL (35%), SSHD (30%).
δ-θ-α-β	δ: CTM-0.4 (75%), CTM-0.5 (75%), CTM-0.3 (70%), STD (65%); θ: SAV (65%), STD (60%), SSHD (55%), SSVL (55%); α: SSHD (75%), STA (65%), SSVL (65%), SCC (50%); β: SAV (60%), SSVL (55%), CTM-0.4 (50%), STD (50%).

**Table 8:** The results of proposed method (SODP + AsyLnCPSO-GA) optimized in the Kaggle, U-Bonn and NSC-ND datasets.

Dataset	The Proportion of Features in *gBest*	Classification Accuracy		
	δ-θ-α-β	Min	Max	Avg
Kaggle (Dog)	δ: CTM-0.5 (90%), CTM-0.4 (85%), SAV (55%), CTM-0.3 (55%); θ: SSVL (85%), SCC (85%), STD (70%), SAV (65%); α: CTM-0.5 (65%), SSVL (55%), STD (55%), SSHD (45%); β: CTM-0.5 (75%), CTM-0.3 (75%), SSVL (75%), STD (65%);	0.9584	0.9714	0.9612
Kaggle (Human)	δ: CTM-0.4 (90%), CTM-0.5 (80%), SSHD (55%), SCC (55%); θ: STD (70%), CTM-0.3 (65%), CTM-0.5 (65%), SSVL (55%); α: SDC (80%), SAV (55%), STD (50%), CTM-0.4 (50%); β: STA (90%), STD (80%), SAV (70%), SSVL (70%);	0.9273	0.9455	0.9371
U-Bonn	δ: SSHD (95%), STA (75%), SDC (65%), SCC (65%); θ: CTM-0.3 (80%), SSHD (75%), SSVL (70%), SCC (55%); α: STD (75%), SDC (70%), SSVL (70%), SSVL (65%); β: STD (70%), CTM-0.5 (70%), SCC (65%), SSVL (60%);	0.9839	0.9936	0.9877
NSC-ND	δ: SSHD (85%), STA (85%), SSHD (60%), SDC (55%); θ: SSHD (60%), SSVL (60%), STA (50%), SAV (50%); α: CTM-0.4 (80%), STA (75%), CTM-0.3 (60%), SSHD (55%); β: STD (60%), CTM-0.3 (60%), CTM-0.4 (60%), SSVL (55%);	0.9921	0.9987	0.9954

**Table 9:** Comparison of the proposed method with the exiting work. AUC: area under the curve; SEN: sensitivity; SPE: specificity; ACC: classification accuracy.

Dataset	Method	Features	Subjects	Classifier	Cross Validation	Performance
Kaggle	STW [52]	Correlation coefficient	5 dogs, 2 patients	SVM	10	Dog: 0.9432 (AUC) Human: 0.9349 (AUC)
	ACS [53]	Spectral power, correlation between channels	4 dogs, 8 patients	Random forest	Leave-one-out	Dog: 0.9651 (ACC) Human: 0.9172 (ACC)
	EMD [54]	Statistical and spectral moments	5 dogs, 2 patients	MAML	10	Dog: 0.9563 (ACC) Human: 0.9528 (ACC)
	mRMR-GA [55]	Decorrelation time, Hjorth parameters, etc.	5 dogs, 2 patients	SVM	5	Dog: 0.9028 (SEN) Human: 0.8853 (SEN)
	Proposed method	Geometric features	4 dogs, 2 patients	Bayesian	10	Dog: 0.9714 (ACC) Human: 0.9455 (ACC)
U-Bonn	RKHS [56]	Covariance descriptors	Set A–E	SR	10	Set D, E: 0.9888 (ACC)
	MMSFL-OWFB [57]	Kraskov entropy	Set A–E	SVM	10	0.992 (ACC)
	Niobium [58]	Euclidean distance, cosine distance, etc.	Set A–E	SVM	5	Set D, E: 0.96 (ACC)
	EMD [59]	IMFs	Set A–E	MLPNN	10	0.952(ACC)
	Proposed method	Geometric features	Set D, E	Bayesian	10	Set D, E: 0.9936 (ACC)
NSC-ND	RKHS [56]	Covariance descriptors	10 patients	SR	10	0.9848 (ACC)
	SDE [60]	Parameters of fitted histograms	10 patients	SVM	10	0.991 (ACC)
	MMSFL-OWFB [57]	Kraskov entropy	10 patients	SVM	10	0.98 (ACC)
	ED [61]	Signal data density	10 patients	1-NN	10	0.9897 (ACC)
	Proposed method	Geometric features	10 patients	Bayesian	10	0.9987 (ACC)
CHB-MIT	EMD [62]	Mean of joint instantaneous amplitude, etc.	24 patients	RF, Bayesian	10	0.9941 (RF, ACC); 0.9516 (Bayesian, ACC)
	WPD [63]	Wavelet coefficients, etc.	24 patients	ANFIS	10	0.9404 (ACC)
	DWT [64]	Sigmoid entropy, etc.	23 patients	SVM	10	0.9421 (SEN)
	AM-FBC [65]	Matrix determinant, etc.	23 patients	SVM	Leave-one-out	0.99 (SEN) 0.89 (SPE)
	Proposed method	Geometric features	23 patients	Bayesian	10	0.9535 (ACC)

## Data Availability

(1) Boston Children’s Hospital and the Massachusetts Institute of Technology scalp EEG dataset (CHB-MIT): https://archive.physionet.org/physiobank/database/chbmit/ (accessed on 15 July 2022). (2) Kaggle dataset: https://www.kaggle.com/competitions/seizure-detection/data (accessed on 16 October 2022). (3) U-Bonn dataset: https://www.upf.edu/web/ntsa/downloads (accessed on 16 October 2022). (4) NSC-ND dataset: https://www.researchgate.net/publication/308719109_EEG_Epilepsy_Datasets (accessed on 16 October 2022).

## Abbreviations of Terms

**Abbreviation**	**Description**
EEG	Electroencephalography
SODP	Second-order differential plot
GA	Genetic algorithm
PSO	Particle swarm optimization
AsyLnCPSO	PSO with asynchronous learning factor
STD	Standard descriptor
SAV	Sum of the angles between consecutive vectors
SSHD	Sum of the shortest distance of each point from the 45-degree line
STA	Sum of the triangle area using consecutive vectors
CTM	Central tendency measure
SDC	Sum of distances to coordinate
SSVL	Sum of successive vectors length
SCC	Sum of the centroid-to-centroid distance of successive triangles

## Appendix A. Description of the GA, PSO and AsyLnCPSO Algorithms

### Appendix A.1. Genetic Algorithm (GA)

GA transmits population (particles) through the three operators of selection, crossover and mutation for population renewal. First, the roulette strategy is used in the selection. Secondly, discrete crossover is applied such that each variable of the child is randomly selected to cross over according to the variable value of the parent individual, with equal probability of forming a new child and crossover rate of 0.5. Finally, Gaussian mutation is employed, which changes the value of each gene in each chromosome, with a mutation rate of 0.01, as follows [66]:

(A1) $$x_{id}^{t}=x_{id}^{t}+N_{id}^{t}(0,\sigma )$$

where $N_{id}^{t}\left(0,\sigma \right)$ is a standard normally distributed random value of the $i^{th}$ chromosome in iteration t.

### Appendix A.2. Particle Swarm Optimization (PSO)

In the PSO algorithm, each solution is referred to as a particle, and each particle is distributed in a position to follow the current optimal particles in the multidimensional problem space flight to find the optimal solution [67]. Moreover, the fitness function measures each movement of particles and calculates the corresponding value to evaluate the particle’s position, i.e., the fitness value, according to which the flight of the particle changes. The position and velocity of particles can be updated as follows:

(A2) $$\begin{array}{l} v_{id}^{t+1}=w\cdot v_{id}^{t}+c_{1}\cdot \psi_{1}\cdot \left(pBest_{id}^{t}-x_{id}^{t}\right)+c_{2}\cdot \psi_{2}\cdot \left(gBest_{d}^{t}-x_{id}^{t}\right)\\ x_{id}^{t+1}=x_{id}^{t}+v_{id}^{t+1} \end{array}$$

where $x_{id}^{t+1}$ and $v_{id}^{t+1}$ are the position and velocity, respectively, of the $i^{th}$ particle in iteration t+1; $c_{1}$ and $c_{2}$ are the positive acceleration coefficients (in this paper $c_{1}=c_{2}=2$); $pbest_{id}^{t}$ is the best position of the $i^{th}$ particle and $gbest_{d}^{t}$ is the best position of all particles in iteration t; $\psi_{1}$ and $\psi_{2}$ are uniformly distributed random coefficients in interval [0,1]; and w is an inertia weight parameter (here, w=0.7).

### Appendix A.3. Asynchronous Learning Factor Changes of PSO (AsyLnCPSO)

To avoid the algorithm of PSO falling into the local optimum, asynchronous learning factors $c_{1}$ and $c_{2}$ are introduced in the PSO, namely AsyLnCPSO. The mathematical formulae of $c_{1}$ and $c_{2}$ are defined as follows:

(A3) $$c_{1}=c_{1\_\max}-\frac{c_{1\_\max}-c_{1\_\min}}{t_{\max}}\times t$$

(A4) $$c_{2}=c_{2\_\max}-\frac{c_{2\_\max}-c_{2\_\min}}{t_{\max}}\times t$$

where, $c_{1\_\max}=3,\;c_{1\_\min}=0.5$ and $c_{2\_\max}=3,\;c_{2\_\min}=1$, $t_{\max}^{}$ represent the maximum number of iterations. In the process of optimization, each learning factor depends on itself. Particles have high social learning ability and low self-learning ability at the beginning of optimization. On the contrary, particles have low social learning ability and high self-learning ability at the end of optimization [35].

## Appendix B. Extra Tables

**Table A1:** Mean ± standard deviation and one-way ANOVA results (F-Value and *p*-Value) of STD–CTM-0.5 in the δ, θ, α and β frequency bands.

	δ Frequency Band				θ Frequency Band			
Feature	Seizure	Seizure-Free	F-Value	*p*-Value	Seizure	Seizure-Free	F-Value	*p*-Value
STD	10.68 ± 10.66	2.626 ± 7.62	85.472	9.454 × 10^−19^	91.66 ± 172.09	19.98 ± 79.76	37.062	2.4 × 10^−8^
SAV	727.74 ± 385.36	227.05 ± 167.5	320.896	1.454 × 10^−54^	3106.43 ± 2827.25	908.09 ± 815.07	159.752	5.67 × 10^−26^
SDC	20115.8 ± 10368.1	6682.9 ± 4630.7	316.268	5.663 × 10^−54^	26917.5 ± 24837.9	908.09 ± 815.07	172.344	1.24 × 10^−25^
STA	6.3249 ± 6.502	1.548 ± 4.698	80.123	9.296 × 10^−18^	1249.8 ± 2318.1	269.47 ± 1077.14	33.044	1.512 × 10^−8^
SSHD	734.79 ± 391.5	228.44 ± 168.35	319.049	2.5 × 10^−54^	3264.8 ± 3015.5	941.19 ± 844.78	154.255	1.122 × 10^−25^
SCC	1252.1 ± 667.25	389.1 ± 286.8	318.951	2.573 × 10^−54^	5591.3 ± 5164.8	1611.7 ± 1446.9	154.138	1.126 × 10^−25^
SSVL	1474.24 ± 785.4	458.3 ± 337.7	319.061	2.491 × 10^−54^	6635.6 ± 6128.6	1912.9 ± 1717.1	154.149	1.117 × 10^−25^
CTM-0.3	0.619 ± 0.19	0.928 ± 0.069	525.113	1.393 × 10^−77^	0.86 ± 0.186	0.991 ± 0.029	211.460	5.377 × 10^−23^
CTM-0.4	0.708 ± 0.178	0.957 ± 0.048	408.887	3.736 × 10^−65^	0.903 ± 0.153	0.993 ± 0.026	137.766	5.638 × 10^−17^
CTM-0.5	0.773 ± 0.161	0.971 ± 0.037	323.897	6.052 × 10^−55^	0.93 ± 0.125	0.994 ± 0.024	96.527	2.511 × 10^−13^
	**α Frequency Band**				**β Frequency Band**			
**Feature**	**Seizure**	**Seizure-Free**	**F-Value**	***p*-Value**	**Seizure**	**Seizure-Free**	**F-Value**	***p*-Value**
STD	56.996 ± 97.713	12.81 ± 48.52	32.280	2.453 × 10^−9^	1156.2 ± 2083.7	327.1 ± 835.6	30.832	4.818 × 10^−8^
SAV	2071.1 ± 1631.3	629.57 ± 527.9	126.153	1.502 × 10^−31^	13547.4 ± 12057.4	5610.7 ± 4671.2	85.142	1.088 × 10^−18^
SDC	28810.4 ± 22047.6	8580.12 ± 7112.2	124.175	1.471 × 10^−33^	64792.5 ± 59132.5	25392.3 ± 20699.6	89.382	1.806 × 10^−19^
STA	215.2 ± 395.9	48.92 ± 179.9	33.244	1.664 × 10^−8^	170741 ± 307823	52644 ± 123649	28.643	1.389 × 10^−7^
SSHD	2142.5 ± 1736.4	641.1 ± 536.59	124.421	1.167 × 10^−30^	16434.1 ± 14936.7	6777.1 ± 5677.7	82.541	3.298 × 10^−18^
SCC	3661.6 ± 2968.9	1095.3 ± 917.2	124.411	1.219 × 10^−30^	27275 ± 24803	11195.7 ± 9358.4	83.143	2.55 × 10^−18^
SSVL	4321.1 ± 3502.5	1293.5 ± 1082.1	124.431	1.214 × 10^−30^	34545.9 ± 31394.8	14276.9 ± 11977.1	82.233	3.762 × 10^−18^
CTM-0.3	0.793 ± 0.192	0.982 ± 0.033	109.004	1.514 × 10^−39^	0.811 ± 0.21	0.959 ± 0.063	102.430	7.895 × 10^−22^
CTM-0.4	0.858 ± 0.165	0.989 ± 0.029	75.944	6.15 × 10^−28^	0.864 ± 0.180	0.973 ± 0.048	77.163	3.327 × 10^−17^
CTM-0.5	0.898 ± 0.141	0.992 ± 0.026	56.941	9.065 × 10^−21^	0.898 ± 0.154	0.981 ± 0.038	61.583	3.124 × 10^−14^

**Table A2:** Mean ± standard deviation, one-way ANOVA results (F-Value and *p*-Value) and classification accuracy (ACC) of time-domain features: root mean square ~ clearance indicator in the δ frequency band.

	Time-Domain Feature (δ Frequency Band)				
Feature	Seizure	Seizure-Free	F-Value	*p*-Value	ACC
Root Mean Square	55.057 ± 27.982	20.794 ± 13.312	273.3	1.011 × 10^−48^	0.8031
Peak–Peak Value	545.311 ± 234.13	265.673 ± 113.991	260.61	1.4 × 10^−46^	0.7972
Skewness	75139.13 ± 725886.695	31472.762 ± 63317.906	0.8116	0.368	0.5984
Kurtosis	4.306 ± 1.588	6.373 ± 2.863	90.005	1.389 × 10^−19^	0.6902
Shape Indicator	1.32 ± 0.079	1.413 ± 0.116	98.715	3.656 × 10^−21^	0.6817
Crest Indicator	3.245 ± 0.408	3.817 ± 0.585	144.977	3.881 × 10^−29^	0.7025
Impulse Indicator	4.346 ± 0.84519	5.574 ± 1.413	125.735	6.68 × 10^−26^	0.6989
Clearance Indicator	5.343 ± 1.279	7.199 ± 2.289	113.119	1.017 × 10^−23^	0.6874
